# Structure and Function of the Type III Secretion System of *Pseudomonas aeruginosa*

**DOI:** 10.2174/138920312804871210

**Published:** 2012-12

**Authors:** Marlies Galle, Isabelle Carpentier, Rudi Beyaert

**Affiliations:** 1 Department of Biomedical Molecular Biology, Ghent University, Technologiepark 927, B-9052 Ghent, Belgium; the; 2 Department for Molecular Biomedical Research, Unit of Molecular Signal Transduction in Inflammation, VIB, Technologiepark 927, B-9052 Ghent, Belgium

**Keywords:** Disease, immunity, immune evasion, infection, Pseudomonas aeruginosa, therapy, Type III secretion system

## Abstract

*Pseudomonas aeruginosa* is a dangerous pathogen particularly because it harbors multiple virulence factors. It causes several types of infection, including dermatitis, endocarditis, and infections of the urinary tract, eye, ear, bone, joints and, of particular interest, the respiratory tract. Patients with cystic fibrosis, who are extremely susceptible to Pseudomonas infections, have a bad prognosis and high mortality. An important virulence factor of *P. aeruginosa*, shared with many other gram-negative bacteria, is the type III secretion system, a hollow molecular needle that transfers effector toxins directly from the bacterium into the host cell cytosol. This complex macromolecular machine works in a highly regulated manner and can manipulate the host cell in many different ways. Here we review the current knowledge of the structure of the *P. aeruginosa* T3SS, as well as its function and recognition by the immune system. Furthermore, we describe recent progress in the development and use of therapeutic agents targeting the T3SS.

## INTRODUCTION

*Pseudomonas aeruginosa* is a gram-negative bacterium that causes a wide variety of acute and chronic infections. Patients with cystic fibrosis are extremely susceptible to *P. aeruginosa* infections [1]. It is also a frequent cause of acute pneumonia, especially in patients who are mechanically ventilated, immunocompromized, or have underlying diseases such as HIV or cancer [2]. The type III secretion system (T3SS), a major virulence determinant that manipulates eukaryotic host cell responses, is present in a broad range of pathogens. It is a specialized needle-like structure that delivers effector toxins directly from the bacteria into the host cytosol in a highly regulated manner. This system is activated on contact with eukaryotic cell membranes, interferes with signal transduction, and causes cell death or alterations in host immune responses.

## STRUCTURE OF THE *PSEUDOMONAS AERUGINOSA* T3SS

The T3SS is evolutionarily connected with the flagellar system, but which one developed first is not clear [3]. The T3SS is a complex macromolecular machine that is structurally and functionally conserved. Five major families of the T3SS can be distinguished [4, 5]: the Ysc-family, the Inv-Mxi-Spa family, and the Ssa-Esc family of animal pathogens, and two different Hrp T3SS of plant pathogens. The Yop system of *Yersinia* spp, the Asc system of *Aeromonas salmonicida*, and the Psc system of *P. aeruginosa* are examples of Ysc types. The Inv-Mxi-Spa family includes the *Salmonella enterica* (SPI-1) and *Shigella* systems, and the Ssa-Esc family includes the systems of *S. enterica* (SPI-2) and of enteropathogenic *E. coli* (EPEC) and enterohaemorrhagic *E*.* coli* (EHEC). The similarities found between these families are not based on their sequences but on their functions, which reflects the ability of pathogenic bacteria to adapt specifically to their environment. The first discovery of the existence of a T3SS in *P. aeruginosa* was in 1996 [6] but it took till 2005 before the T3SS was structurally visualised [7].

**Fig. (1):**
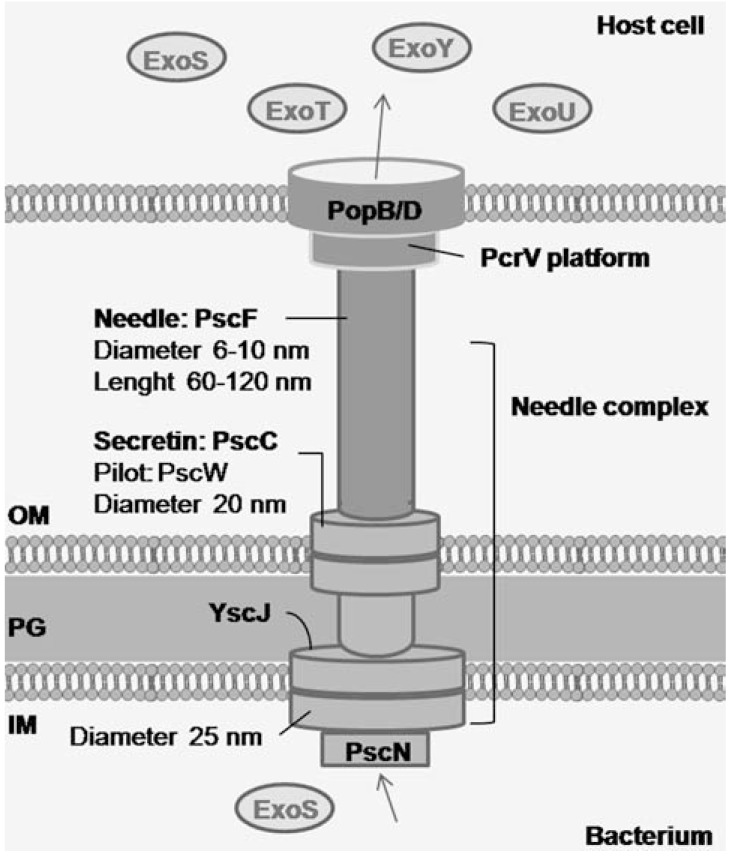
Structure of the *P. aeruginosa* T3SS. The T3SS consists of a needle complex, translocating apparatus, and effector toxins that are translocated directly from the bacterium to the host cell cytosol. This process is facilitated by chaperones and regulatory proteins.

The T3SS of *P. aeruginosa* consists of several proteins that form a macromolecular complex spanning the inner bacterial membrane, the periplasmic space, the peptidoglycan layer, the outer bacterial membrane, the extracellular space, and the host cell membrane (Fig. **1**). At the functional level one can define the secretion apparatus or needle complex, the translocation or targeting apparatus, and the secreted toxins (effector proteins) and cognate chaperones [8].

### The Secretion Apparatus or Needle Complex

The secretion apparatus of *P. aeruginosa* consists of a basal body and a needle-like structure or injectisome. The basal body of the T3SS, in which the needle-like structure is anchored, is the only intracellular part of the T3SS and spans the inner bacterial membrane, the peptidoglycan layer, and the outer bacterial membrane [9]. The basal body brings effector toxins from the bacterial cytosol to the needle-like structure. It consists of an inner membrane component and an outer membrane component. While the inner membrane component of the T3SS is predicted to contain PscJ, the outer membrane component consists of the PscC protein of the secretin family of outer membrane proteins, which function as the outer membrane component of a variety of multicomponent export systems, such as Type II secretion systems and Type IV pilus biogenesis. Secretins form large pores by the assembly of 12-14 units into a homomultimeric ring structure that spans the outer membrane and protrudes into the periplasm [8]. Likewise, PscC oligomerizes and together with the lipoprotein PscW forms a channel in the outer membrane [10]. Other proteins, such as lipoprotein PscJ [11] and ATPase PscN [12], probably form part of the basal body of the T3SS of *P. aeruginosa*, but this has not been confirmed yet. 

The needle-like structure or injectisome is assembled by helical polymerization of PscF proteins [7, 13]. It resembles a hollow molecular needle that brings effector toxins from the basal body to the translocator apparatus. It is also thought to be involved in sensing host cells, which is needed to activate the T3SS.

### The Translocation or Targeting Apparatus

The translocation apparatus is responsible for transporting the effector toxins from the needle-like structure across the host cell plasma membrane and delivering it into the host cell cytosol. In non-secreting conditions the T3SS is build up until the needle-like structure with a low basal secretion of effector proteins into the surrounding, however insufficient for intoxication of cells. Upon contact with a host cell, an active injectisome is formed, ending with a functional translocation apparatus that is inserted in the host cell plasma membrane, forming a functional translocation pore. The T3SS translocation apparatus of *P. aeruginosa* needs three proteins: PopB, PopD and PcrV [14], which are collectively known as translocators. These proteins are secreted by the T3SS itself, they interact with each other, and they are needed to form a translocation pore [15]. PopB and PopD, which consist of hydrophobic domains, oligomerize to form the translocation pore. PcrV is not a part of the pore itself and is hydrophilic, though its presence is required to form a functional translocation pore [16-18].

### The Secreted Effector Toxins

**Fig. (2):**
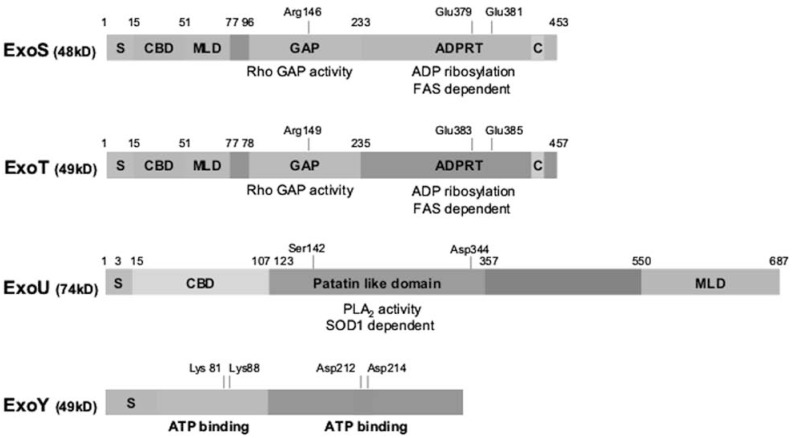
Structure of ExoS, ExoT, ExoY and ExoU of *P. aeruginosa*. *P. aeruginosa* ExoS and ExoT are bifunctional Type III–secreted cytotoxins. Both have an S-domain, CBD, MLD, and C-domain, and GAP and ADPRT activities. ExoS requires Arg146 for the GAP activity, and both Glu379 and Glu381 are required for ADPRT activity. Arg149 is required for the GAP activity of ExoT, and residues Glu383 and Glu385 are crucial for its ADPRT activity. ExoU contains a patatin-like domain that is necessary for phospholipase A2 activity. Residues Ser142 and Asp344 are required for this activity. ExoY is a 378-amino acid adenylyl cyclase. Residues Lys81, Lys88, Asp212 and Asp214 are required for its activity and are thought to be necessary for interactions between ExoY and ATP. S: secretion signal, CBD: chaperone binding domain, MLD: membrane localization domain, GAP: GTPase activating protein, ADPRT: ADP ribosyl transferase; C: cofactor binding site.

So far four effector toxins of *P. aeruginosa* have been identified: ExoS, ExoT, ExoU and ExoY. The four effector toxins are rarely all present in one strain and different strains have either the ExoS or the ExoU gene [19]. Expression of T3SS effector toxins is correlated with clinical disease outcome [20] and the effector toxins present determine the phenotype of a strain. Whereas ExoS causes apoptotic cell death [21], ExoU causes cell lysis [22]. ExoT and ExoS share 76% amino acid identity [13] and have many similarities, but they are also very different [23]. ExoT and ExoS are both bifunctional proteins with an N-terminal GTPase-activating (GAP) domain and a C-terminal adenosine diphosphate ribosyltransferase domain (ADPRT-domain) (Fig. **2**). ExoS is an enzyme of 453 amino acids. Its first 15 N-terminal residues constitute the secretion domain, residues 15-51 the chaperone binding domain, and residues 51-72 the membrane localization domain. The secretion signal directs effector toxins to the T3SS apparatus. The chaperone binding domain binds SpcS, which is important for the secretion of ExoS, probably by maintaining ExoS in a secretion-competent conformation [24]. The membrane localization domain targets the effector toxins to the plasma membrane of the host cell, which is needed for efficient modification of host proteins. The GAP domain with Rho GAP activity is between residues 96 and 233, just before the ADPRT domain with ADP ribosylating activity, which is at residues 233-453. Within the ADPRT domain, residues 418-429 make up the binding site for cofactor FAS, which is a 14-3-3 protein needed for ADPRT activity. Glu379 and Glu381 are also crucial for ADPRT activity [25]. Arg146, within the GAP domain, is required for GAP activity.

ExoT is an enzyme of 457 amino acids, resembling ExoS in structure. Within the first 50 residues it has an N-terminal secretion domain, a chaperone binding domain, and a membrane localization domain. Residues 78-235 contain the GAP domain, and the ADPRT domain is located at residue 235-457. The cofactor binding site lies between residues 422-433. As in ExoS, Arg149 is required for the GAP activity of ExoT [26] and residues Glu383 and Glu385 are required for ADP ribosylation activity [25]. 

ExoU is a 687-amino acid phospholipase with phospholipase A2 activity, which requires residues Ser142 and Asp344 [27]. The N-terminal secretion domain is assumed to be within the first 15 residues of the protein, and the chaperone binding domain lies between residues 3 and 123 [28]. This is followed by a patatin-like domain, between residues 107 and 357, which contains the phospholipase A2 activity [29]. Between residues 357 and 550 there is no recognizable motif, but between residues 550–687 there is a membrane localization domain [30]. 

ExoY is a secreted adenylyl cyclase [31] of 378 amino acids. It has two domains that act together to bind ATP. Its activity relies on residues Lys81, Lys88, Asp212 and Asp214 which are also thought to represent the ATP binding site. 

Secretion and translocation signals have not been identified in the T3SS effectors, which makes the identification of novel virulence factors very difficult. Bioinformatics analysis of a large number of genome sequences of plant and animal pathogens are being used to identify candidate T3SS effector proteins [32].

### Chaperones of the T3SS Apparatus

The chaperones of the T3SS apparatus are small bacterial cytosolic proteins that assist the assembly and operation of the T3SS. Although they also assist the secreted effector toxins, they are never exported through the T3SS needle. These chaperones can be divided into different classes depending on which type of protein they assist: class I chaperones assist the pore forming proteins, class II chaperones the subunits of the needle-like structure, and class III chaperones the effector toxins. Different functions and action mechanisms of chaperones of the T3SS have been suggested [33]. The helical components of the injectisome, for instance, are chaperoned by class I chaperones that maintain the components in a monomeric state by binding the subunits and masking the domains involved in polymerization to prevent the subunits from associating with each other. They might also assist in the delivery of proteins to the export channel. The needle component PscF is kept in a 1:1:1 conformation in the cytosol by the chaperones PscE and PscG [34]. The toxicity of the hydrophobic translocators is neutralized by class II chaperones [35]. The presence of free translocator-chaperones might signal the bacterium that the pore is made and the injectisome is functional [36].

The translocator proteins PopB and PopD are chaperoned by PcrH [37]. The effector proteins have dedicated chaperones belonging to class I and interacting with their cognate partner through the chaperone binding domain. ExoS and ExoT are chaperoned by SpcS, which is required for maximum secretion of these proteins, and SpcU is a chaperone for ExoU, but no chaperone for ExoY has been identified. All these chaperones can help to sequester their partners in the bacterial cytosol to inhibit inappropriate secretion, but they also facilitate the appropriate delivery of their cognate partner for secretion [38]. There is also some evidence that they are important for targeting their partners to ATPase and thereby facilitate their unfolding [39].

## REGULATION OF THE T3SS PROTEINS

### Regulation of Transcription and Secretion of the T3SS Effector Toxins

The biogenesis of the T3SS injectisome and the translocation of effector toxins is tightly controlled and is dependent on about 36 genes encoded in five clustered operons in the *P. aeruginosa* chromosome [40]. In addition, at least six other genes located around this chromosome are important in the biogenesis of the T3SS. 

The signal that triggers the expression of T3SS genes is commonly known as direct host cell contact sensed by the T3SS needle, but the exact signaling mechanism is not known. Metabolic stress reduces the ability of *P. aeruginosa* to secrete proteins by decreasing the percentage of bacterial cells that can assemble functional secretion systems [41, 42]. The secretion itself is regulated at two levels: transcription of T3SS genes and secretion of the proteins. Transcription itself is regulated by different regulatory proteins that are substrates of the T3SS for secretion. By removing activators or suppressors that regulate the transcription of the T3SS genes through secretion, the regulation of transcription of T3SS genes is coupled to the initiation of the actual secretion itself [43, 44], as transcription is induced upon activation of secretion [45, 46].

**Fig. (3):**
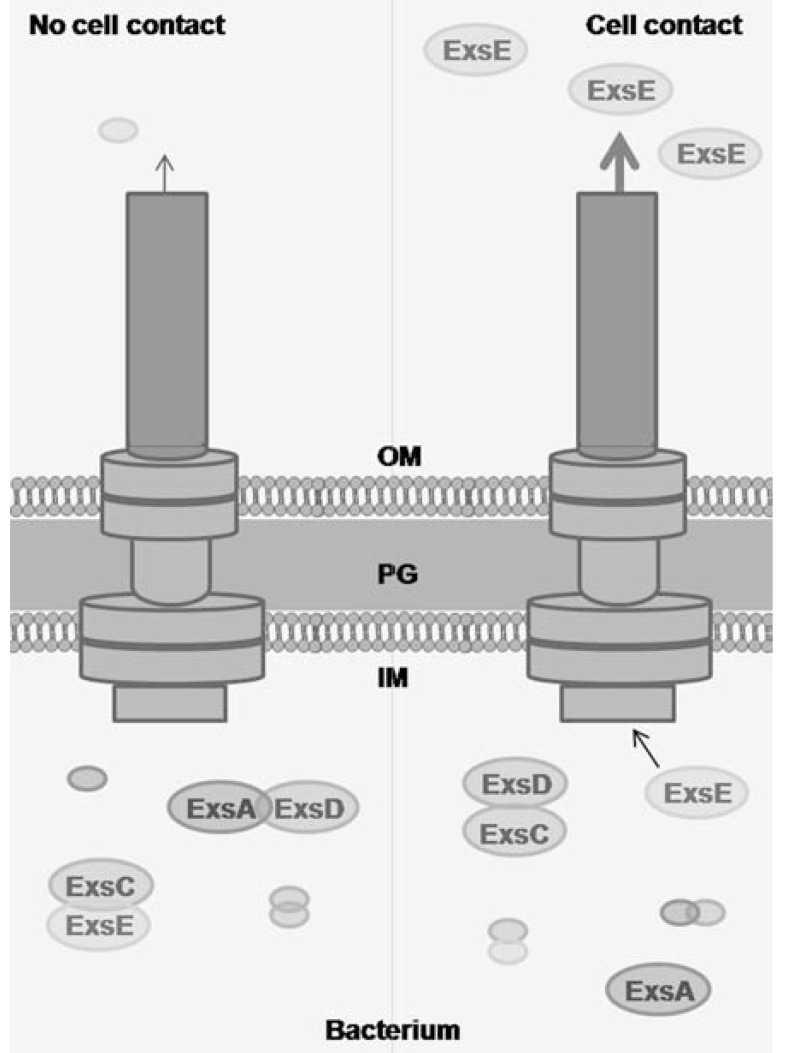
Coupling of T3SS transcription and secretion. Coupling of transcription and secretion occurs by the interaction of four proteins: ExsA, ExsD, ExsC and ExsE. In non-secreting conditions, ExsA is bound to ExsD, which inhibits transcriptional activation of ExsA. ExsC is bound to ExsE. Once secretion is activated, ExsE is exported, which allows ExsC to bind ExsD and inactivate its inhibitory activity. This allows ExsA to bind to the promoter region of T3SS genes.

The transcription of *P. aeruginosa* T3SS genes is regulated by the transcriptional activator ExsA, which binds to an ExsA consensus element in the promoter of T3SS genes and to its own promoter [47]. The secretion activity is coupled to ExsA dependent transcription by three proteins: ExsC, ExsD and ExsE (Fig. **3**). In non-secreting conditions, ExsA dependent transcription is inhibited by binding to the anti-activator ExsD [45]. The anti-activator ExsC can bind and negatively regulate ExsD [48], but in non-secreting conditions it is bound to its higher binding affinity partner, ExsE [43, 44]. During secreting conditions, when the T3SS injectisome is activated upon host cell contact, the coupling of transcription and secretion is achieved by the export of ExsE into the host cell cytosol [49]. The reduced levels of ExsE liberate the ExsC chaperone that binds ExsD. ExsD subsequently frees ExsA and activates transcription. A more detailed overview of regulation of the T3SS can be found in [46, 50]. 

The T3SS effector protein ExoS prevents triggering of type III secretion by bacteria when they are attached to cells, which points to feedback regulation. There is evidence that translocation of ExoS is self-regulated and that this inhibition of translocation can be achieved by either its GAP or ADPRT enzymatic activities [51]. It has also been proposed that a complex of PcrV and PcrG in the bacterial cytosol [52] and the bacterial protein PopN [53] play roles in protein secretion. However, this stands in contrast to the findings of Schoehn *et al.* [37], who could not show a role of these proteins in protein secretion. It has also been suggested that once the effector toxins are released, regulatory mechanisms ‘reset’ the T3SS and ‘re-load’ the T3SS machine with new effector toxins [54]. 

In almost all existing T3SS the total levels of each of the effector toxins, needed to intoxicate the cell, is related to the increased levels of protein expression for the structural components of the injectisome. However, in the regulation of the T3SS of *P. aeruginosa*, this is not always the case. It has been proposed that the system might have initially evolved to protect the organism from predators that naturally inhabit the soil and water [55], such as either free living [56] or biofilm associated amoeba [57]. This notion is supported by the presence of a functional T3SS in most isolates of environmental *P. aeruginosa* [58]. The broad conservation of different targets in eukaryotic organisms can explain why the T3SS resulted in a system that is also active against humans. In addition, this broad conservation of targets could also explain why *P. aeruginosa* has only four effector toxins, which is fewer than that those of other organisms expressing T3SS.

### Assembly of the T3SS Machine and Pore Formation

It is not only the T3SS protein secretion and transcription that is regulated, but also the assembly of the T3SS machine and needle length. Moreover, PscN, an ATPase that provides energy for the T3SS, is controlled by PscL [59]. Before the injectisome secretes effector toxins, it exports its own distal components. Based on the similarity between the T3SS system and the flagellar system, it has been suggested that the T3SS machine is assembled sequentially. The distal structures of the injectisome grow by polymerization of the subunits at the tip of the nascent structure and there is no checkpoint between the assembly of the rod and the needle. The needle length is strictly regulated and there are different models explaining the control of the needle length. Based on the studies of its homolog YscP in* Yersinia*, PscP has been suggested to act as a molecular ruler in *P. aeruginosa* [reviewed by [9]]. 

**Fig. (4):**
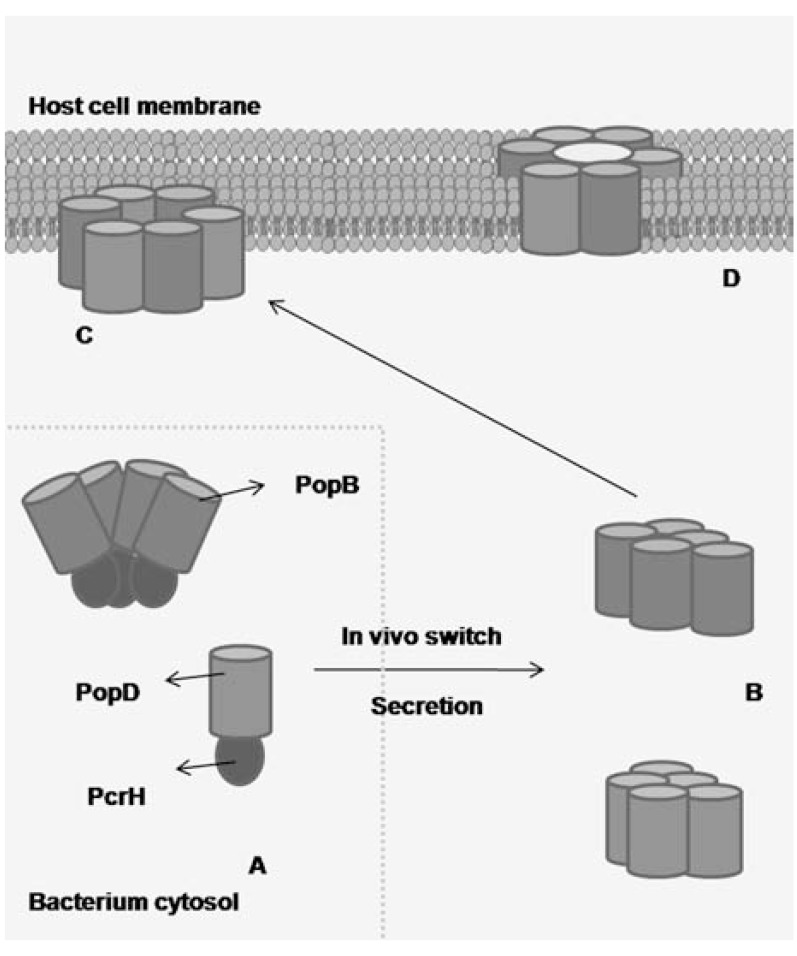
Pore formation by PopB and PopD in *P. aeruginosa*. (A) PcrH binds to PopB and PopD in the bacterial cytosol and thereby prevents their aggregation and/or activation. (B) Upon the ‘*in vivo* switch’, which may involve recognition of the T3SS secretion or transport through the needle, PopB and PopD form metastable oligomers. (C) PopB and PopD might associate into a homomeric and/or heteromeric structures that recognize microdomain lipid rafts on the plasma membrane of target cells and form a ring-like structure. (D) They form a channel on the target membrane that allows transport of other T3SS proteins. The molecules are shown as pentamers only for schematic purposes.

Once the needle is assembled, the T3SS switches substrate specificity, and the formation of the translocation pore and export of effector toxins is triggered by contact with the host cell. Both PopB and PopD are chaperoned by PcrH in the bacterial cytosol. *In vitro*, recombinant PopB and PopD dissociate from their cognate chaperone in acidic conditions and oligomerize to form soluble complexes, either as stable heterodimers or as metastable heterooligomers. In the presence of liposomes, the monomers form metastable oligomers that subsequently can bind and disrupt cholesterol-rich membranes generating the ring for the translocation pore. In the case of the PopB/PcrH complex, this occurs within a pH range of 5±7, while PopD is only oligomeric at acidic pH and in the absence of PcrH [37]. Briefly, in the model proposed to explain how PopB and PopD form the translocon (Fig. **4**), these two proteins are bound to their chaperone PcrH in the bacterial cytosol, which prevents aggregation or activation of the subunits. Upon activation by an *in vivo* switch, PopB and PopD are released from their chaperone and form metastable oligomers. Then PopB and PopD can form homomeric or heteromeric structures that recognize microdomain lipid rafts on the plasma membrane of target cells. This is followed by the formation of ring-like structures with an outer and inner diameter of 80 Å and 40 Å, respectively. This so-called PopB/PopD translocon forms a channel in the target membrane that allows transport of other components of the *P. aeruginosa* T3SS from the bacterial into the host cell cytosol. 

As mentioned above, all three translocator proteins have to be present to form a functional translocation pore. PcrV is secreted and serves as a molecular platform at the tip of the needle, where PopB and PopD oligomerize. Insertion of PopD, but not PopB, requires the needle tip protein PcrV [18, 60]. Surprisingly, PcrV cannot bind artificial liposomes [18] and so far no interaction has been shown between PopB/D and PcrV [51]. It is possible that there is a weak affinity between them, needing multiple simultaneous interactions or a bridging protein to connect both translocators. It is also not known if oligomerization of PopB and PopD and formation of the pore is facilitated by the PcrV platform, or whether the PcrV platform is linked with PopB and PopD after pore formation [61].

## FUNCTION OF THE T3SS

### Function of Effector Toxins

The *P. aeruginosa* T3SS has been shown to be a major reason for the cytotoxicity of this bacterium for a wide range of host cells [55, 62-64]. Comparison of the relative contributions of ExoU, ExoS and ExoT effector toxins to mortality, bacterial persistence in the lung, and dissemination in a mouse model of acute pneumonia indicated that secretion of ExoU had the greatest impact, ExoS had an intermediate effect, and ExoT had a minor effect [19]. 

As described above, ExoS and ExoT have both a GAP and an ADPRT domain. The GAP activities of ExoS and ExoT are biochemically and biologically identical: they inactivate several Rho GTPases by bringing them from an active GTP bound state in an inactive GDP-bound state. Both ExoS [65, 66] and ExoT [67, 68] target the small GTPases Rho, Rac and Cdc42, which maintain organization of the host cell actin skeleton. Inactivating these GTPases leads to disruption of the actin skeleton [66], which manifests itself in cell rounding and detachment, and inhibition of cell migration and phagocytosis, as shown by the decrease in internalization of *P. aeruginosa* by certain cell types [26, 69, 70]. In addition, the GAP activity of ExoT also inhibits cytokinesis [71].

Although the GAP activities of ExoT and ExoS share the same targets, their ADPRT activities are different. ExoS ADPRT activity mediates cytotoxicity while ExoT interferes with host cell phagocytic activity. ExoT ADP-ribosylates phosphoglycerate kinase and CrkI/II proteins, which signal upstream of RhoGTPases [72]. The ADPRT domain of ExoT cooperates with the GAP domain to disrupt the actin cytoskeleton. It is also involved in delayed wound healing [73], which allows the opportunistic pathogen to invade through breaches in the mucosal barriers. Moreover, the ExoT ADPRT domain causes apoptotic cell death, probably by inhibiting Crk [74]; this apoptosis occurs later than in ExoS induced apoptosis [75]. ExoS ADP ribosylates a diverse group of proteins, such as cyclophilin A, IgG3, apolipoprotein A1, vimentin [76] and members of the Ras-like GTPase superfamily [77, 78]. ExoS also ADP ribosylates members of the ezrin/radixin/moesin family [79, 80], which are important in the reorganization of the actin cytoskeleton. The ADP ribosylation of all these proteins results in actin cytoskeleton disruption, apoptotic-like cell death, and inhibition of DNA synthesis, endocytosis and vesicular trafficking. Increased levels of ExoS correlate with increased *in vitro* cytotoxicity [81] and pulmonary damage in animal models and cystic fibrosis patients [40, 82, 83]. Apart from the ADPRT domain, also the membrane localization domain and the cofactor binding site of ExoS and ExoT are important for their ADP ribosylating function. The presence of a host cell cofactor, first identified as factor associating ExoS (FAS) but now known as a 14-3-3 protein, is absolutely required for ADPRT activity of ExoS and ExoT, which implies that their ADPRT activity is inhibited until they are injected in the host cell cytosol to prevent them from harming the bacteria. The membrane localization domain was shown to localize ExoS to the host cell membrane, where it ADP ribosylates RAS, and also to localize ExoS to the early endosomes, the late endosomes, and finally the Golgi apparatus [84]. 

ExoS not only has a T3SS contact-dependent intracellular function in cytotoxicity but can also interact as a soluble extracellular protein with T cells and monocytes [85, 86]. This results in T cell apoptosis and proinflammatory cytokine and chemokine production by activated monocytes [87, 88]. ExoS has been reported to activate monocytic cells via a MyD88-dependent pathway, using both TLR2 and TLR4. Interestingly, the TLR2 activity was localized to the C-terminal domain of ExoS while the TLR4 activity was localized to the N-terminal domain [89]. In contrast, we previously showed that T3SS-mediated injection of ExoS in macrophages negatively regulates *P. aeruginosa* induced proteolytic maturation and secretion of the proinflammatory cytokine IL-1β [90], indicating that ExoS has different immune regulatory functions dependent on its location. Moreover ExoS deficiency also switched the mode of macrophage death from apoptosis to pro-inflammatory pyroptosis [90].

ExoU is a potent cytotoxin whose host cellular targets and mechanism of action are not completely known. It is a member of the phospholipase A family, possessing phospholipase A2 activity with broad substrate specificity [91]. ExoU activity is correlated with acute cytotoxicity in epithelial cells and macrophages, contributes to injury in mouse models of pneumonia [22], and is associated with severe human *P. aeruginosa* infectious diseases where ExoU specifically kills neutrophils. Depletion of neutrophils leads to an immunosuppressive effect that makes the host more susceptible to secondary infections [92, 93]. This modulation of the host immune response and its ability to induce extensive tissue destruction explains why ExoU, together with ExoS, has a prominent role in *P. aeruginosa* pathogenicity. Besides its predominantly cytotoxic activities, ExoU also activates c-jun N-terminal kinase and AP-1 mediated pro-inflammatory gene expression in epithelial cells [94], but inhibits caspase-1-mediated pro-inflammatory cytokine production in macrophages [95]. 

Like ExoS and ExoT, ExoU needs a cofactor for its activity. In contrast to ExoS and ExoT, which need 14-3-3 as cofactor, ExoU uses superoxide dismutase, though its enzymatic activity is not required [96, 97]. Like ExoS and ExoT, ExoU also has a membrane localization domain that is needed for efficient activity. After secretion into the host cell cytosol, ExoU associates with the membrane, which brings the effector close to its possible target, a host cell factor that activates the phospholipases activity of the patatin-like domain [30]. Another interesting aspect of ExoU is its ubiquitination within the host cell. However, this modification does not lead to ExoU degradation and its biological role remains unclear [98]. 

ExoY, the most recently discovered effector protein, has not been implicated directly in cellular toxic effects and its role remains unclear. However, ExoY was shown to be toxic in yeast cells [99] and to induce cell rounding in eukaryotic immune cells [100]. It is an adenylate cyclase that elevates the intracellular cAMP levels in cultured mammalian cells and causes actin cytoskeleton reorganization. Like the other effector toxins, ExoY needs a cofactor for its activity, but its identity is unknown [31].

### Function of the Needle and Translocation Apparatus

The most prominent function of the T3SS machine is the transport of effector toxins from the bacterium into the host cell cytosol. This transport is very efficient because less than 0.1% of the effector toxins escape into the extracellular space [101]. Pore formation by the injectisome was first shown for *Yersinia* spp. in erythrocytes, which are not a natural target for T3SS but are suitable for studying pore formation [9]. The translocator apparatus is situated at the interface between pathogen and host cell and plays a crucial role in transport of effector toxins across the plasma membrane of the host cell. Interestingly, the *P. aeruginosa* PopB and PopD translocator proteins can functionally complement yopB and yopD translocator mutants of *Yersinia pseudotuberculosis*, which demonstrates functional similarity in the infection processes of this organism and *P. aeruginosa*. However, efficient complementation also requires PcrV, which is also part of the translocator apparatus [102, 103]. 

The role of the T3SS is more than just transport of effector toxins. Though much of the T3SS mediated damage is probably caused by the translocated effectors, there is increasing evidence that insertion of the needle complex itself can contribute to host cell injury. This was elegantly demonstrated by whole-genome microarray comparison of the transcriptional responses of epithelial cells to *P. aeruginosa* wild type and isogenic mutants [104, 105]. Similarly, we previously showed that the *P. aeruginosa* translocation pore can trigger caspase-1 activation in macrophages independently of any of the known T3SS effector proteins [106]. Caspase-1 mediates the proteolytic maturation of IL-1β and also results in pyroptotic cell death [95, 106]. This is consistent with previous reports that suggested pyroptotic host cell death depends on the T3SS needle but not on effector toxins [107-109]. A similar caspase-1 activating function has been reported for the T3SS of *Yersinia* [110]. The activation of caspase-1 by *P. aeruginosa* requires the intracellular Nod-like receptor Ipaf (also known as NLRC4) [95, 111, 112], which recognizes the T3SS in two ways: indirectly by detecting flagellin, and directly by detecting the basal body rod component PscI of the T3SS apparatus [113]. Similarly, Ipaf also detects the rod proteins of *Salmonella typhimurium*, *Burkholderia pseudomallei*, *Escherichia coli*, and *Shigella flexneri*. All these proteins share a sequence motif with the *Pseudomonas *rod protein PscI and flagellin which are both essential for detection by Ipaf. Flagellin and rod proteins are believed to be unintentionally delivered to the mammalian cytosol through the T3SS. 

## THE T3SS AND DISEASE

*P. aeruginosa* causes serious, often antibiotic resistant infections in immunocompromised individuals, burn victims and patients requiring mechanical ventilation [114, 115]. For example, once *P. aeruginosa* is established in the airways of cystic fibrosis patients, it is almost impossible to eradicate and it frequently leads to death [116]. Although T3SS is not necessary for infection, its importance in virulence has been well established in animal models of chronic infections [117] and acute lung infections, which showed that the T3SS is associated with higher mortality rates [118], [119] and that the presence of a functional T3SS in *P. aeruginosa* infecting humans is associated with severe disease and bad prognosis [20, 120]. In clinical studies of lower respiratory tract infections, the relative risk of mortality was six-fold higher in acute infections associated with expression of the T3SS proteins ExoS, ExoT, ExoU, or PcrV [120]. Furthermore, T3SS-expressing isolates are predominantly found in acute infections rather than in patients with cystic fibrosis, which indicates that T3SS is a detrimental virulence factor particularly in acute infections.

**Fig. (5):**
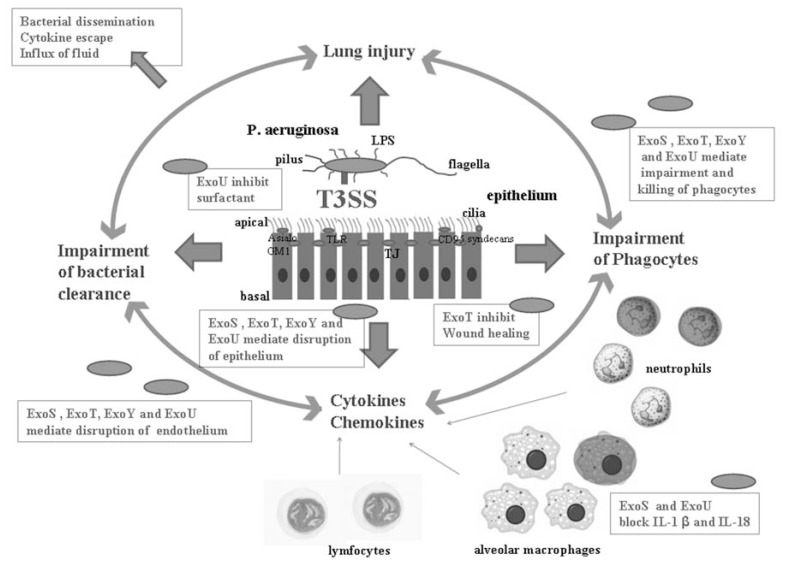
Role of the T3SS in pathogenicity and host responses to *P. aeruginosa* acute lung infection. The T3SS creates a self-propagating pathogenic cycle. T3SS induced acute lung injury is connected with T3SS induced enhancement of proinflammatory mediators, such as cytokines and chemokines, and a decrease in the number of alveolar phagocytes. Both of these lead to impairment of bacterial clearance from the lungs and a higher mortality rate. The darkly stained phagocytes are killed by a T3SS dependent mechanism. The airway epithelial cells, macrophages, neutrophils, and lymphocytes release mediators that enable the host to mount a response to the invading bacteria. The T3SS facilitates tissue injury and invasion of the pathogen by different mechanisms. ExoS, ExoT, ExoY and ExoU induce cell death of epithelial cells, ExoT inhibits wound healing, and ExoU inhibits surfactant production. Together, these lead to disruption of the alveolar epithelium. ExoU and ExoS can block the production of IL-1β and IL-18 induced by the T3SS needle and diminish the early inflammatory response. However, the inflammatory response is often excessive and induces lung injury. ExoS, ExoT, ExoY and ExoU mediate the impairment and killing of phagocytes, which impairs bacterial clearance. ExoS, ExoT, ExoY and ExoU mediate the disruption of both the epithelial and the endothelial barriers, which leads to dissemination of the pathogen, escape of cytokines into the systemic circulation, and influx of protein rich fluids. The consequence is septic bacteremia and shock.

Epithelial cells, endothelial cells, lymphocytes, neutrophils and macrophages, as well as their targeting by the T3SS, all play important roles during *P. aeruginosa* infection (Fig. **5**). The normal healthy airway epithelium is extremely resistant to *P. aeruginosa* infection, which also explains its opportunistic character. This resistance is due to the mucosal barrier, mucociliary clearance, tight junctions [121], and numerous signal cascades that lead to expression of mucines [122], antimicrobial peptides [123] and chemokines [124]. This in turn leads to the recruitment of phagocytes and inflammatory mediators. Upon infection, airway cells induce signaling components to their apical surface to initiate inflammatory responses [125]. Disrupted epithelium is much more susceptible to *P. aeruginosa* infection, and damaged airway epithelium has increased amounts of the receptor asialoGM1 on the basolateral side of the epithelial cell to which the pathogen can bind [126]. Cytotoxic bacteria expressing T3SS attach to the epithelium and inject their cytoskeleton disrupting toxins, which causes the loss of tight junctions and increases the number of cells that expose their basolateral surface [127]. ExoS, ExoT, ExoU and ExoY are not only involved in the loss of integrity of the epithelial barrier but also endothelial barriers [128]. The disruption of these epithelia allows the pathogen to disseminate into the bloodstream and pro-inflammatory mediators to leak into the systemic circulation, which eventually leads to septic shock [129]. Moreover, the inhibition of wound healing by ExoT facilitates invasion and possibly dissemination of the pathogen. Airway epithelial cells, macrophages, neutrophils, and lymphocytes release mediators that enable the host to mount an immune response against the invading bacteria. T3SS enhances the pro-inflammatory response, for example by triggering IL-1β maturation, and this leads to acute lung injury. In the early phase of the inflammatory response, the death or impairment of alveolar neutrophils and macrophages mediated by ExoS, ExoT, ExoU and ExoY leads to impairment of bacterial clearance from the lungs [92]. When *P. aeruginosa* are not eradicated from the airway, there is a greater opportunity for T3SS to produce and/or inject more effector toxins and proteins into the host cells, which results in injury of the pulmonary tissue and the characteristic symptoms of pneumonia induced by* P. aeruginosa*. Thus, T3SS is involved in a self propagating pathogenic circle in which induction of pro-inflammatory mediators, depletion of phagocytes, and impairment of bacterial clearance are connected with *P. aeruginosa* induced lung injury [130]. This also explains why T3SS-expressing bacterial strains are associated with high mortality and bad prognosis. 

## THERAPEUTIC INTERFERENCE WITH THE T3SS

The challenge for the future remains to use the acquired knowledge of the cell biology and the role of the T3SS in host-pathogen interactions to develop better therapeutics for infectious disease. Resistance to antibiotics is a major problem in the treatment of *P. aeruginosa* infections in critically ill patients. As T3SS is unique to the pathogen, it is a convenient target for therapeutic and prophylactic interventions. Unlike conventional antibiotics, targeting a virulence mechanism that is associated almost exclusively with pathogenic bacteria will not perturb the normal body flora. Although T3SS is downregulated in cystic fibrosis patients, in whom *P. aeruginosa* infection contributes to disease severity and mortality, these patients make antibodies against it [131]. This suggests that preventing *P. aeruginosa* possessing an active T3SS from colonizing tissue might be used as early intervention for patients with cystic fibrosis. 

Administration of monoclonal antibodies against T3SS structural proteins has been used to counteract the action of various pathogens. In this context, antibodies against the T3SS needle-tip protein PcrV, which controls T3SS effector secretion, have been shown to be effective against laboratory strains of *P. aeruginosa* in animal models of septic shock [132] and acute and chronic airway infection [133, 134], and have recently entered Phase I/II clinical trials [135]. Use of small molecule inhibitors to directly interfere with T3SS has also become feasible recently. For example, small molecule inhibitors of the phospholipase activity of ExoU and the ADPRT activity of ExoS have been identified, and each one was shown to protect against death in models of *P. aeruginosa* infection [99, 136]. It might also be better to interfere with the formation or activity of the T3SS translocation pore than to target the effectors because T3SS effector independent functions of the T3SS seem to play a key role in bacterial pathogenesis. Recently, five compounds that inhibit the activity of the *P. aeruginosa* T3SS apparatus and the secretion of all its effector proteins were identified in a large scale compound library screen [137]. The molecular targets of these inhibitors are not known, but their activity against the T3SS in three different bacterial species points to a conserved structural protein target. The potential development of these and other T3SS inhibitor compounds will be an area of exciting development in coming years. Finally, since the presence of the T3SS correlates with the progression and outcome of infection, diagnostic identification of T3SS components in clinical isolates might be useful for deciding on how to manage patients.

## CONCLUSIONS AND FUTURE PERSPECTIVES

The last few years have seen remarkable progress in the understanding of the structure and function of the T3SS of *P. aeruginosa* and other pathogenic bacteria. However, much remains to be discovered. For example, we still need a better understanding of the relative contributions of the different bacterial T3SS effectors and the T3SS delivery machine itself, and how they cooperate with one another or with other virulence mechanisms in bacterial pathogenesis. This will require the definition of the physiologically relevant host cell targets, as well as the identification of the physiological role of pore formation. Considering the likely possibility that novel T3SS effector proteins will be identified in the near future by using large scale bioinformatics approaches, the time ahead will be as exciting as the last few years. The ultimate goal is to reveal new general principles of host-pathogen interactions and to provide new targets and strategies for antimicrobial therapies.

## References

[1] Kobayashi H, Kobayashi O, Kawai S (2009). Pathogenesis and clinical manifestations of chronic colonization by Pseudomonas aeruginosa and its biofilms in the airway tract. J. Infect. Chemother.

[2] Trautmann M, Lepper PM, Haller M (2005). Ecology of Pseudomonas aeruginosa in the intensive care unit and the evolving role of water outlets as a reservoir of the organism. Am. J. Infect. Control.

[3] Saier MHJ (2004). Evolution of bacterial type III protein secretion systems. Trends Microbiol.

[4] Troisfontaines P, Cornelis GR (2005). Type III secretion: more systems than you think. Physiology (Bethesda).

[5] Pallen MJ, Chaudhuri RR, Henderson IR (2003). Genomic analysis of secretion systems. Curr. Opin. Microbiol.

[6] Yahr TL, Barbieri JT, Frank DW (1996). Genetic relationship between the 53- and 49-kilodalton forms of exoenzyme S from Pseudomonas aeruginosa. J. Bacteriol.

[7] Pastor A, Chabert J, Louwagie M, Garin J, Attree I (2005). PscF is a major component of the Pseudomonas aeruginosa type III secretion needle. FEMS Microbiol. Lett.

[8] Moraes TF, Spreter T, Strynadka NC (2008). Piecing together the type III injectisome of bacterial pathogens. Curr. Opin. Struct. Biol.

[9] Cornelis GR (2006). The type III secretion injectisome. Nat. Rev. Microbiol.

[10] Burghout P, Beckers F, de Wit E, van Boxtel R, Cornelis GR, Tommassen J, Koster M (2004). Role of the pilot protein YscW in the biogenesis of the YscC secretin in Yersinia enterocolitica. J. Bacteriol.

[11] Burns RE, McDaniel-Craig A, Sukhan A (2008). Site-directed mutagenesis of the Pseudomonas aeruginosa type III secretion system protein PscJ reveals an essential role for surface-localized residues in needle complex function. Microb. Pathog.

[12] Sorg JA, Blaylock B, Schneewind O (2006). Secretion signal recognition by YscN, the Yersinia type III secretion ATPase. Proc. Natl. Acad. Sci. USA.

[13] Yahr TL, Goranson J, Frank DW (1996). Exoenzyme S of Pseudomonas aeruginosa is secreted by a type III pathway. Mol. Microbiol.

[14] Mueller CA, Broz P, Cornelis GR (2008). The type III secretion system tip complex and translocon. Mol. Microbiol.

[15] Sarker MR, Neyt C, Stainier I, Cornelis GR (1998). The Yersinia Yop virulon: LcrV is required for extrusion of the translocators YopB and YopD. J. Bacteriol.

[16] Fields KA, Nilles ML, Cowan C, Straley SC (1999). Virulence role of V antigen of Yersinia pestis at the bacterial surface. Infect. Immun.

[17] Marenne M-Nl, Journet L, Mota LJ, Cornelis GR (2003). Genetic analysis of the formation of the Ysc-Yop translocation pore in macrophages by Yersinia enterocolitica: role of LcrV, YscF and YopN. Microb. Pathog.

[18] Goure J, Pastor A, Faudry E, Chabert J, Dessen A.a, Attree I (2004). The V antigen of Pseudomonas aeruginosa is required for assembly of the functional PopB/PopD translocation pore in host cell membranes. Infect. Immun.

[19] Feltman H, Schulert G, Khan S, Jain M, Peterson L, Hauser AR (2001). Prevalence of type III secretion genes in clinical and environmental isolates of Pseudomonas aeruginosa. Microbiology.

[20] Hauser AR (2009). The type III secretion system of Pseudomonas aeruginosa: infection by injection. Nat. Rev. Microbiol.

[21] Kaufman MR, Jia J, Zeng L, Ha U, Chow M, Jin S (2000). Pseudomonas aeruginosa mediated apoptosis requires the ADP-ribosylating activity of exoS. Microbiology.

[22] Finck-Barbançon V, Goranson J, Zhu L, Sawa T, Wiener-Kronish JP, Fleiszig SM, Wu C, Mende-Mueller L, Frank DW (1997). ExoU expression by Pseudomonas aeruginosa correlates with acute cytotoxicity and epithelial injury. Mol. Microbiol.

[23] Barbieri JT, Sun J (2004). Pseudomonas aeruginosa ExoS and ExoT. Rev. Physiol. Biochem. Pharmacol.

[24] Shen DK, Quenee L, Bonnet M, Kuhn L, Derouazi M, Lamotte D, Toussaint B, Polack B (2008). Orf1/SpcS chaperones ExoS for type three secretion by Pseudomonas aeruginosa. Biomed. Environ. Sci.

[25] Radke J, Pederson KJ, Barbieri JT (1999). Pseudomonas aeruginosa exoenzyme S is a biglutamic acid ADP-ribosyltransferase. Infect. Immun.

[26] Garrity-Ryan L, Kazmierczak B, Kowal R, Comolli J, Hauser A, Engel JN (2000). The arginine finger domain of ExoT contributes to actin cytoskeleton disruption and inhibition of internalization of Pseudomonas aeruginosa by epithelial cells and macrophages. Infect. Immun.

[27] Rabin SDP, Hauser AR (2005). Functional regions of the Pseudomonas aeruginosa cytotoxin ExoU. Infect. Immun.

[28] Finck-Barbançon V, Yahr TL, Frank DW (1998). Identification and characterization of SpcU, a chaperone required for efficient secretion of the ExoU cytotoxin. J. Bacteriol.

[29] Phillips RM, Six DA, Dennis EA, Ghosh P (2003). *In vivo* phospholipase activity of the Pseudomonas aeruginosa cytotoxin ExoU and protection of mammalian cells with phospholipase A2 inhibitors. J. Biol. Chem.

[30] Rabin SDP, Veesenmeyer JL, Bieging KT, Hauser AR (2006). A C-terminal domain targets the Pseudomonas aeruginosa cytotoxin ExoU to the plasma membrane of host cells. Infect. Immun.

[31] Yahr TL, Vallis AJ, Hancock MK, Barbieri JT, Frank DW (1998). ExoY, an adenylate cyclase secreted by the Pseudomonas
aeruginosa type III system. Proc. Natl. Acad. Sci. USA.

[32] Yang Y, Zhao J, Morgan RL, Ma W, Jiang T (2010). Computational prediction of type III secreted proteins from gram-negative bacteria. BMC Bioinformat.

[33] Ghosh P (2004). Process of protein transport by the type III secretion system. Microbiol. Mol. Biol. Rev.

[34] Quinaud M, Chabert J, Faudry E, Neumann E, Lemaire D, Pastor A, Elsen S, Dessen Aa, Attree I (2005). The PscE-PscF-PscG complex controls type III secretion needle biogenesis in Pseudomonas aeruginosa. J. Biol. Chem.

[35] Neyt C, Cornelis GR (1999). Role of SycD, the chaperone of the Yersinia Yop translocators YopB and YopD. Mol. Microbiol.

[36] Page A-L, Parsot C (2002). Chaperones of the type III secretion pathway: jacks of all trades. Mol. Microbiol.

[37] Schoehn G, Di Guilmi AM, Lemaire D, Attree I, Weissenhorn W, Dessen A (2003). Oligomerization of type III secretion proteins PopB and PopD precedes pore formation in Pseudomonas. EMBO J.

[38] Parsot C, Hamiaux C, Page AL (2003). The various and varying roles of specific chaperones in type III secretion systems. Curr. Opin. Microbiol.

[39] Akeda Y, Galán JE (2005). Chaperone release and unfolding of substrates in type III secretion. Nature.

[40] Frank DW (1997). The exoenzyme S regulon of Pseudomonas aeruginosa. Mol. Microbiol.

[41] Rietsch A, Wolfgang MC, Mekalanos JJ (2004). Effect of metabolic imbalance on expression of type III secretion genes in Pseudomonas aeruginosa. Infect. Immun.

[42] Rietsch A, Mekalanos JJ (2006). Metabolic regulation of type III secretion gene expression in Pseudomonas aeruginosa. Mol. Microbiol.

[43] Urbanowski ML, Lykken GL, Yahr TL (2005). A secreted regulatory protein couples transcription to the secretory activity of the Pseudomonas aeruginosa type III secretion system. Proc. Natl. Acad. Sci. USA.

[44] Rietsch A, Vallet-Gely I, Dove SL, Mekalanos JJ (2005). ExsE, a secreted regulator of type III secretion genes in Pseudomonas aeruginosa. Proc. Natl. Acad. Sci. USA.

[45] McCaw ML, Lykken GL, Singh PK, Yahr TL (2002). ExsD is a negative regulator of the Pseudomonas aeruginosa type III secretion regulon. Mol. Microbiol.

[46] Brutinel ED, Yahr TL (2008). Control of gene expression by type III secretory activity. Curr. Opin. Microbiol.

[47] Brutinel ED, Vakulskas CA, Brady KM, Yahr TL (2008). Characterization of ExsA and of ExsA-dependent promoters required for expression of the Pseudomonas aeruginosa type III secretion system. Mol. Microbiol.

[48] Dasgupta N, Lykken GL, Wolfgang MC, Yahr TL (2004). A novel anti-anti-activator mechanism regulates expression of the Pseudomonas aeruginosa type III secretion system. Mol. Microbiol.

[49] Urbanowski ML, Brutinel ED, Yahr TL (2007). Translocation of ExsE into Chinese hamster ovary cells is required for transcriptional induction of the Pseudomonas aeruginosa type III secretion system. Infect. Immun.

[50] Diaz MR, King JM, Yahr TL (2011). Intrinsic and extrinsic regulation of Type III secretion gene expression in Pseudomonas aeruginosa. Front. Microbiol.

[51] Cisz M, Lee PC, Rietsch A (2008). ExoS controls the cell contact-mediated switch to effector secretion in Pseudomonas aeruginosa. J. Bacteriol.

[52] Nanao M, Ricard-Blum S, Di Guilmi AM, Lemaire D, Lascoux D, Chabert J, Attree I, Dessen A (2003). Type III secretion proteins PcrV and PcrG from Pseudomonas aeruginosa form a 1:1 complex through high affinity interactions. BMC Microbiol.

[53] Yang H, Shan Z, Kim J, Wu W, Lian W, Zeng L, Xing L, Jin S (2007). Regulatory role of PopN and its interacting partners in type III secretion of Pseudomonas aeruginosa. J. Bacteriol.

[54] Demers B, Sansonetti PJ, Parsot C (1998). Induction of type III secretion in Shigella flexneri is associated with differential control of transcription of genes encoding secreted proteins. EMBO J.

[55] Pukatzki S, Kessin RH, Mekalanos JJ (2002). The human pathogen Pseudomonas aeruginosa utilizes conserved virulence pathways to infect the social amoeba Dictyostelium discoideum. Proc. Natl. Acad. Sci. USA.

[56] Abd H, Wretlind B, Saeed A, Idsund E, Hultenby K, Sandström G (2008). Pseudomonas aeruginosa utilises its type III secretion system to kill the free-living amoeba Acanthamoeba castellanii. J. Eukaryot. Microbiol.

[57] Matz C, Moreno AM, Alhede M, Manefield M, Hauser AR, Givskov M, Kjelleberg S (2008). Pseudomonas aeruginosa uses type III secretion system to kill biofilm-associated amoebae. ISME J.

[58] Ferguson MW, Maxwell JA, Vincent TS, da Silva J, Olson JC (2001). Comparison of the exoS gene and protein expression in soil and clinical isolates of Pseudomonas aeruginosa. Infect. Immun.

[59] Blaylock B, Riordan KE, Missiakas DM, Schneewind O (2006). Characterization of the Yersinia enterocolitica type III secretion ATPase YscN and its regulator, YscL. J. Bacteriol.

[60] Goure J, Broz P, Attree O, Cornelis GR, Attree I (2005). Protective anti-V antibodies inhibit Pseudomonas and Yersinia translocon assembly within host membranes. J. Infect. Dis.

[61] Mota LJ (2006). Type III secretion gets an LcrV tip. Trends Microbiol.

[62] Hauser AR, Engel JN (1999). Pseudomonas aeruginosa induces type-III-secretion-mediated apoptosis of macrophages and epithelial cells. Infect. Immun.

[63] Kang PJ, Hauser AR, Apodaca G, Fleiszig SM, Wiener-Kronish J, Mostov K, Engel JN (1997). Identification of Pseudomonas aeruginosa genes required for epithelial cell injury. Mol. Microbiol.

[64] Miyata S, Casey M, Frank DW, Ausubel FM, Drenkard E (2003). Use of the Galleria mellonella caterpillar as a model host to study the role of the type III secretion system in Pseudomonas aeruginosa pathogenesis. Infect. Immun.

[65] Pederson KJ, Vallis AJ, Aktories K, Frank DW, Barbieri JT (1999). The amino-terminal domain of Pseudomonas aeruginosa ExoS disrupts actin filaments via small-molecular-weight GTP-binding proteins. Mol. Microbiol.

[66] Goehring UM, Schmidt G, Pederson KJ, Aktories K, Barbieri JT (1999). The N-terminal domain of Pseudomonas aeruginosa exoenzyme S is a GTPase-activating protein for Rho GTPases. J. Biol. Chem.

[67] Kazmierczak BI, Engel JN (2002). Pseudomonas aeruginosa ExoT acts *in vivo* as a GTPase-activating protein for RhoA, Rac1, and Cdc42. Infect. Immun.

[68] Krall R, Schmidt G, Aktories K, Barbieri JT (2000). Pseudomonas aeruginosa ExoT is a Rho GTPase-activating protein. Infect. Immun.

[69] Frithz-Lindsten E, Du Y, Rosqvist R, Forsberg A (1997). Intracellular targeting of exoenzyme S of Pseudomonas aeruginosa via type III-dependent translocation induces phagocytosis resistance, cytotoxicity and disruption of actin microfilaments. Mol. Microbiol.

[70] Cowell BA, Chen DY, Frank DW, Vallis AJ, Fleiszig SM (2000). ExoT of cytotoxic Pseudomonas aeruginosa prevents uptake by corneal epithelial cells. Infect. Immun.

[71] Shafikhani SH, Engel J (2006). Pseudomonas aeruginosa type III-secreted toxin ExoT inhibits host-cell division by targeting cytokinesis at multiple steps. Proc. Natl. Acad. Sci. USA.

[72] Sun J, Barbieri JT (2003). Pseudomonas aeruginosa ExoT ADP-ribosylates CT10 regulator of kinase (Crk) proteins. J. Biol. Chem.

[73] Garrity-Ryan L, Shafikhani S, Balachandran P, Nguyen L, Oza J, Jakobsen T, Sargent J, Fang X, Cordwell S, Matthay MA, Engel JN (2004). The ADP ribosyltransferase domain of Pseudomonas aeruginosa ExoT contributes to its biological activities. Infect. Immun.

[74] Cho SY, Klemke RL (2000). Extracellular-regulated kinase activation and CAS/Crk coupling regulate cell migration and suppress apoptosis during invasion of the extracellular matrix. J. Cell Biol.

[75] Shafikhani SH, Morales C, Engel J (2008). The Pseudomonas aeruginosa type III secreted toxin ExoT is necessary and sufficient to induce apoptosis in epithelial cells. Cell. Microbiol.

[76] Coburn J, Dillon ST, Iglewski BH, Gill DM (1989). Exoenzyme S of Pseudomonas aeruginosa ADP-ribosylates the intermediate filament protein vimentin. Infect. Immun.

[77] Coburn J, Gill DM (1991). ADP-ribosylation of p21ras and related proteins by Pseudomonas aeruginosa exoenzyme S. Infect. Immun.

[78] Jansson AL, Yasmin L, Warne P, Downward J, Palmer RH, Hallberg B (2006). Exoenzyme S of Pseudomonas aeruginosa is not able to induce apoptosis when cells express activated proteins, such as Ras or protein kinase B/Akt. Cell. Microbiol.

[79] Maresso AW, Baldwin MR, Barbieri JT (2004). Ezrin/radixin/moesin proteins are high affinity targets for ADP-ribosylation by Pseudomonas aeruginosa ExoS. J. Biol. Chem.

[80] Maresso AW, Deng Q, Pereckas MS, Wakim BT, Barbieri JT (2007). Pseudomonas aeruginosa ExoS ADP-ribosyltransferase inhibits ERM phosphorylation. Cell. Microbiol.

[81] Sawa T, Ohara M, Kurahashi K, Twining SS, Frank DW, Doroques DB, Long T, Gropper MA, Wiener-Kronish JP (1998). *In vitro* cellular toxicity predicts Pseudomonas aeruginosa virulence in lung infections. Infect. Immun.

[82] Liu S, Kulich SM, Barbieri JT (1996). Identification of glutamic acid 381 as a candidate active site residue of Pseudomonas aeruginosa exoenzyme S. Biochemistry.

[83] Liu S, Yahr TL, Frank DW, Barbieri JT (1997). Biochemical relationships between the 53-kilodalton (Exo53) and 49-kilodalton (ExoS) forms of exoenzyme S of Pseudomonas aeruginosa. J. Bacteriol.

[84] Pederson KJ, Krall R, Riese MJ, Barbieri JT (2002). Intracellular localization modulates targeting of ExoS, a type III cytotoxin, to eukaryotic signalling proteins. Mol. Microbiol.

[85] Bruno TF, Woods DE, Storey DG, Mody CH (1999). Recombinant Pseudomonas exoenzyme S and exoenzyme S from Pseudomonas aeruginosa DG1 share the ability to stimulate T lymphocyte proliferation. Can. J. Microbiol.

[86] Epelman S, Neely GG, Ma LL, Gjomarkaj M, Pace E, Melis M, Woods DE, Mody CH (2002). Distinct fates of monocytes and T cells directly activated by Pseudomonas aeruginosa exoenzyme S. J. Leukoc. Biol.

[87] Bruno TF, Woods DE, Mody CH (2000). Exoenzyme S from Pseudomonas aeruginosa induces apoptosis in T lymphocytes. J. Leukoc. Biol.

[88] Epelman S, Bruno TF, Neely GG, Woods DE, Mody CH (2000). Pseudomonas aeruginosa exoenzyme S induces transcriptional expression of proinflammatory cytokines and chemokines. Infect. Immun.

[89] Epelman S, Stack D, Bell C, Wong E, Neely GG, Krutzik S, Miyake K, Kubes P, Zbytnuik LD, Ma LL, Xie X, Woods DE, Mody CH (2004). Different domains of Pseudomonas aeruginosa exoenzyme S activate distinct TLRs. J. Immunol.

[90] Galle M, Schotte P, Haegman M, Wullaert A, Yang HJ, Jin S, Beyaert R (2008). The Pseudomonas aeruginosa Type III secretion system plays a dual role in the regulation of caspase-1 mediated IL-1beta maturation. J. Cell. Mol. Med.

[91] Sitkiewicz I, Stockbauer KE, Musser JM (2007). Secreted bacterial phospholipase A2 enzymes: better living through phospholipolysis. Trends Microbiol.

[92] Diaz MH, Shaver CM, King JD, Musunuri S, Kazzaz JA, Hauser AR (2008). Pseudomonas aeruginosa induces localized immunosuppression during pneumonia. Infect. Immun.

[93] El Solh AA, Akinnusi ME, Wiener-Kronish JP, Lynch SV, Pineda LA, Szarpa K (2008). Persistent infection with Pseudomonas aeruginosa in ventilator-associated pneumonia. Am. J. Respir. Crit. Care Med.

[94] Cuzick A, Stirling FR, Lindsay SL, Evans TJ (2006). The type III pseudomonal exotoxin U activates the c-Jun NH2-terminal kinase pathway and increases human epithelial interleukin-8 production. Infect. Immun.

[95] Sutterwala FS, Mijares LA, Li L, Ogura Y, Kazmierczak BI, Flavell RA (2007). Immune recognition of Pseudomonas aeruginosa mediated by the IPAF/NLRC4 inflammasome. J. Exp. Med.

[96] Sato H, Feix JB, Frank DW (2006). Identification of superoxide dismutase as a cofactor for the pseudomonas type III toxin, ExoU. Biochemistry.

[97] Schmalzer KM, Benson MA, Frank DW Activation of ExoU phospholipase activity requires specific C-terminal regions. J. Bacteriol.

[98] Stirling FR, Cuzick A, Kelly SM, Oxley D, Evans TJ (2006). Eukaryotic localization, activation and ubiquitinylation of a bacterial type III secreted toxin. Cell. Microbiol.

[99] Arnoldo A, Curak J, Kittanakom S, Chevelev I, Lee VT, Sahebol-Amri M, Koscik B, Ljuma L, Roy PJ, Bedalov A, Giaever G, Nislow C, Merrill AR, Lory S, Stagljar I (2008). Identification of small molecule inhibitors of Pseudomonas aeruginosa exoenzyme S using a yeast phenotypic screen. PLoS Genet.

[100] Cowell BA, Evans DJ, Fleiszig SMJ (2005). Actin cytoskeleton disruption by ExoY and its effects on Pseudomonas aeruginosa invasion. FEMS Microbiol. Lett.

[101] Sundin C, Thelaus J, BrÃ¶ms JE, Forsberg A (2004). Polarisation of type III translocation by Pseudomonas aeruginosa requires PcrG, PcrV and PopN. Microb. Pathog.

[102] Frithz-Lindsten E, HolmstrÃ¶m A, Jacobsson L, Soltani M, Olsson J, Rosqvist R, Forsberg A (1998). Functional conservation of the effector protein translocators PopB/YopB and PopD/YopD of Pseudomonas aeruginosa and Yersinia pseudotuberculosis. Mol. Microbiol.

[103] Bröms JE, Forslund A-L, Forsberg A, Francis MS (2003). PcrH of Pseudomonas aeruginosa is essential for secretion and assembly of the type III translocon. J. Infect. Dis.

[104] Ichikawa JK, English SB, Wolfgang MC, Jackson R, Butte AJ, Lory S (2005). Genome-wide analysis of host responses to the Pseudomonas aeruginosa type III secretion system yields synergistic effects. Cell. Microbiol.

[105] McMorran B, Town L, Costelloe E, Palmer J, Engel J, Hume D, Wainwright B (2003). Effector ExoU from the type III secretion system is an important modulator of gene expression in lung epithelial cells in response to Pseudomonas aeruginosa infection. Infect. Immun.

[106] Galle M, Jin S, Bogaert P, Haegman M, Vandenabeele P, Beyaert R (2012). The Pseudomonas aeruginosa Type III Secretion System Has an Exotoxin S/T/Y Independent Pathogenic Role during Acute Lung Infection. PLoS One.

[107] Dacheux D, Attree I, Toussaint B (2001). Expression of ExsA in trans confers type III secretion system-dependent cytotoxicity on noncytotoxic Pseudomonas aeruginosa cystic fibrosis isolates. Infect. Immun.

[108] Lee VT, Smith RS, Tümmler B, Lory S (2005). Activities of Pseudomonas aeruginosa effectors secreted by the Type III secretion system *in vitro* and during infection. Infect. Immun.

[109] Vance RE, Rietsch A, Mekalanos JJ (2005). Role of the type III secreted exoenzymes S, T, and Y in systemic spread of Pseudomonas aeruginosa PAO1 *in vivo*. Infect. Immun.

[110] Shin H, Cornelis GR (2007). Type III secretion translocation pores of Yersinia enterocolitica trigger maturation and release of pro-inflammatory IL-1beta. Cell. Microbiol.

[111] Franchi L, Stoolman J, Kanneganti TD, Verma A, Ramphal R, Núnez G (2007). Critical role for Ipaf in Pseudomonas aeruginosa-induced caspase-1 activation. Eur. J. Immunol.

[112] Miao EA, Ernst RK, Dors M, Mao DP, Aderem A (2008). Pseudomonas aeruginosa activates caspase 1 through Ipaf. Proc. Natl. Acad. Sci. USA.

[113] Miao EA, Mao DP, Yudkovsky N, Bonneau R, Lorang CG, Warren SE, Leaf IA, Aderem A (2010). Innate immune detection of the type III secretion apparatus through the NLRC4 inflammasome. Proc. Natl. Acad. Sci. USA.

[114] Garau J, Gomez L (2003). Pseudomonas aeruginosa pneumonia. Curr. Opin. Infect. Dis.

[115] Valencia M, Torres A (2009). Ventilator-associated pneumonia. Curr. Opin. Crit. Care.

[116] Lyczak JB, Cannon CL, Pier GB (2002). Lung infections associated with cystic fibrosis. Clin. Microbiol. Rev.

[117] Kukavica-Ibrulj I, Levesque RC (2008). Animal models of chronic lung infection with Pseudomonas aeruginosa: useful tools for cystic fibrosis studies. Lab. Anim.

[118] Faure K, Sawa T, Ajayi T, Fujimoto J, Moriyama K, Shime N, Wiener-Kronish JP (2004). TLR4 signaling is essential for survival in acute lung injury induced by virulent Pseudomonas aeruginosa secreting type III secretory toxins. Respir. Res.

[119] Luna CM, Sibila O, Agusti C, Torres A (2009). Animal models of ventilator-associated pneumonia. Eur. Respir. J.

[120] Roy-Burman A, Savel RH, Racine S, Swanson BL, Revadigar NS, Fujimoto J, Sawa T, Frank DW, Wiener-Kronish JP (2001). Type III protein secretion is associated with death in lower respiratory and systemic Pseudomonas aeruginosa infections. J. Infect. Dis.

[121] Knowles MR, Boucher RC (2002). Mucus clearance as a primary innate defense mechanism for mammalian airways. J. Clin. Invest.

[122] Li JD, Dohrman AF, Gallup M, Miyata S, Gum JR, Kim YS, Nadel JA, Prince A, Basbaum CB (1997). Transcriptional activation of mucin by Pseudomonas aeruginosa lipopolysaccharide in the pathogenesis of cystic fibrosis lung disease. Proc. Natl. Acad. Sci. USA.

[123] Singh PK, Jia HP, Wiles K, Hesselberth J, Liu L, Conway BA, Greenberg EP, Valore EV, Welsh MJ, Ganz T, Tack BF, McCray PBJ (1998). Production of beta-defensins by human airway epithelia. Proc. Natl. Acad. Sci. USA.

[124] DiMango E, Ratner AJ, Bryan R, Tabibi S, Prince A (1998). Activation of NF-kappaB by adherent Pseudomonas aeruginosa in normal and cystic fibrosis respiratory epithelial cells. J. Clin. Invest.

[125] Soong G, Reddy B, Sokol S, Adamo R, Prince A (2004). TLR2 is mobilized into an apical lipid raft receptor complex to signal infection in airway epithelial cells. J. Clin. Invest.

[126] Fleiszig SM, Evans DJ, Do N, Vallas V, Shin S, Mostov KE (1997). Epithelial cell polarity affects susceptibility to Pseudomonas aeruginosa invasion and cytotoxicity. Infect. Immun.

[127] Saiman L, Prince A (1993). Pseudomonas aeruginosa pili bind to asialoGM1 which is increased on the surface of cystic fibrosis epithelial cells. J. Clin. Invest.

[128] Kudoh I, Wiener-Kronish JP, Hashimoto S, Pittet JF, Frank D (1994). Exoproduct secretions of Pseudomonas aeruginosa strains influence severity of alveolar epithelial injury. Am. J. Physiol.

[129] Kurahashi K, Kajikawa O, Sawa T, Ohara M, Gropper MA, Frank DW, Martin TR, Wiener-Kronish JP (1999). Pathogenesis of septic shock in Pseudomonas aeruginosa pneumonia. J. Clin. Invest.

[130] Ader F, Le Berre R, Faure K, Gosset P, Epaulard O, Toussaint B, Polack B, Nowak E, Viget NB, Kipnis E, Guery BP (2005). Alveolar response to Pseudomonas aeruginosa: role of the type III secretion system. Infect. Immun.

[131] Corech R, Rao A, Laxova A, Moss J, Rock MJ, Li Z, Kosorok MR, Splaingard ML, Farrell PM, Barbieri JT (2005). Early immune response to the components of the type III system of Pseudomonas aeruginosa in children with cystic fibrosis. J. Clin. Microbiol.

[132] Shime N, Sawa T, Fujimoto J, Faure K, Allmond LR, Karaca T, Swanson BL, Spack EG, Wiener-Kronish JP (2001). Therapeutic administration of anti-PcrV F(ab')(2) in sepsis associated with Pseudomonas aeruginosa. J. Immunol.

[133] Faure K, Fujimoto J, Shimabukuro DW, Ajayi T, Shime N, Moriyama K, Spack EG, Wiener-Kronish JP, Sawa T (2003). Effects of monoclonal anti-PcrV antibody on Pseudomonas aeruginosa-induced acute lung injury in a rat model. J. Immune Based Ther. Vaccines.

[134] Imamura Y, Yanagihara K, Fukuda Y, Kaneko Y, Seki M, Izumikawa K, Miyazaki Y, Hirakata Y, Sawa T, Wiener-Kronish JP, Kohno S (2007). Effect of anti-PcrV antibody in a murine chronic airway Pseudomonas aeruginosa infection model. Eur. Respir. J.

[135] François B, Luyt CE, Dugard A, Wolff M, Diehl JL, Jaber S, Forel JM, Garot D, Kipnis E, Mebazaa A, Misset B, Andremont A, Ploy MC, Jacobs A, Yarranton G, Pearce T, Fagon JY, Chastre J (2012). Safety and pharmacokinetics of an anti-PcrV PEGylated monoclonal antibody fragment in mechanically ventilated patients colonized with Pseudomonas aeruginosa: A randomized, double-blind, placebo-controlled trial. Crit. Care Med.

[136] Reiniger N, Lee MM, Coleman FT, Ray C, Golan DE, Pier GB (2007). Resistance to Pseudomonas aeruginosa chronic lung infection requires cystic fibrosis transmembrane conductance regulator-modulated interleukin-1 (IL-1) release and signaling through the IL-1 receptor. Infect. Immun.

[137] Aiello D, Williams JD, Majgier-Baranowska H, Patel I, Peet NP, Huang J, Lory S, Bowlin TL, Moir DT (2010). Discovery and characterization of inhibitors of Pseudomonas aeruginosa type III secretion. Antimicrob. Agents Chemother.

